# Weather-Based Prediction Models for the Prevalence of Dengue Vectors *Aedes aegypti* and *Ae. albopictus*

**DOI:** 10.1155/2022/4494660

**Published:** 2022-12-27

**Authors:** J. M. Manel K. Herath, Hemalika T. K. Abeyasundara, W. A. Priyanka P. De Silva, Thilini C. Weeraratne, S. H. P. Parakrama Karunaratne

**Affiliations:** ^1^Entomological Surveillance Unit, Office of Regional Director of Health Services, Kurunegala, Sri Lanka; ^2^Postgraduate Institute of Science, University of Peradeniya, Peradeniya, Sri Lanka; ^3^Department of Statistics and Computer Science, University of Peradeniya, Peradeniya, Sri Lanka; ^4^Department of Zoology, Faculty of Science, University of Peradeniya, Peradeniya, Sri Lanka

## Abstract

Dengue is an important vector-borne disease transmitted by the mosquitoes *Aedes aegypti* and *Ae. albopictus*. In the absence of an effective vaccine, vector control has become the key intervention tool in controlling the disease. Vector densities are significantly affected by the changing weather patterns of a region. The present study was conducted in three selected localities, i.e., urban Bandaranayakapura, semiurban Galgamuwa, and rural Buluwala in the Kurunegala district of Sri Lanka to assess spatial and temporal distribution of dengue vector mosquitoes and to predict vector prevalence with respect to changing weather parameters. Monthly ovitrap surveys and larval surveys were conducted from January to December 2019 and continued further in the urban area up to December 2021. *Aedes aegypti* was found moderately in the urban area and to a lesser extent in semiurban but not in the rural area. *Aedes albopictus* had the preference for rural over urban areas. *Aedes aegypti* preferred indoor breeding, while *Ae. albopictus* preferred both indoor and outdoor. For *Ae. albopictus*, ovitrap index (OVI), premise index (PI), container index (CI), and Breteau index (BI) correlated with both the rainfall (RF) and relative humidity (RH) of the urban site. Correlations were stronger between OVI and RH and also between BI and RF. Linear regression analysis was fitted, and a prediction model was developed using BI and RF with no lag period (*R*^2^ (sq) = 86.3%; *F* = 53.12; *R*^2^ (pred) = 63.12%; model: Log10 (BI) = 0.153 + 0.286^*∗*^ Log10 (RF); RMSE = 1.49). Another prediction model was developed using OVI and RH with one month lag period (*R*^2^ (sq) = 70.21%; *F* = 57.23; model: OVI predicted = 15.1 + 0.528^*∗*^ Lag 1 month RH; RMSE = 2.01). These two models can be used to monitor the population dynamics of *Ae. albopictus* in urban settings to predict possible dengue outbreaks.

## 1. Introduction

Dengue is a fast-growing mosquito borne disease, found especially in the countries located within the equatorial zone, and has been ranked as one of the top ten threats to global health in 2019 by the World Health Organization [1, 2]. It is an arboviral disease caused by infection of one or more of the four dengue virus serotypes. Dengue virus is transmitted from human to human through the bites of infective females of *Aedes aegypti* Linnaeus and *Ae. albopictus* Skuse mosquitoes (Diptera: Culicidae) [3, 4]. *Aedes aegypti* is mainly endophilic and considered the primary vector of the disease. It has adapted to human habitats and breeds primarily in artificial water containers such as water storage barrels, old tires, and flower pots. *Aedes albopictus* is mainly exophilic, less adapted to human habitats, and mainly breeds in natural containers such as tree stumps and coconut shells and to a lesser extent in artificial containers [5–7]. Although *Ae. aegypti* has a higher distribution range and a higher disease transmission capacity, *Ae. albopictus* is considered one of the most invasive mosquito species emerging as a global public-health threat [8].

In Sri Lanka, the prevalence of high-risk level dengue is mainly due to favorable meteorological conditions which promote high vector abundance. Previous workers have shown unique distribution patterns of the two vector species in different geographical regions of the country. *Aedes aegypti* is the predominant *Aedes* species in northern [9] and western [10] provinces, whereas *Ae. albopictus* is dominant in western, north-western, central, and southern provinces [11–13]. Since an effective vaccine against the virus is not available, dengue prevention and control mainly depend on effective vector control measures against immature aquatic stages and adults. Many studies have shown that the creation of new larval habitats, as a result of rapid urbanization programs, has resulted in the expansion of dengue vector populations into new areas playing a significant role in vector distribution patterns [6, 14, 15]. Therefore, urbanization substantially increases adult mosquito density, larval development rate, and adult survival time of the vector species which, in turn, potentially increase disease transmission.

In Sri Lanka, dengue has been reported as a public health problem since the 1960s. A major upward shift in the disease incidence has been reported since 2009. Sri Lanka faced a massive dengue epidemic in 2017, with 186,101 reported cases and 440 deaths [16]. Kurunegala district, situated in the north-western province of Sri Lanka, annually reports >2000 dengue cases, and dengue fever outbreaks have frequently occurred in the district since 2014 (Epidemiological data, 2020). As in several other districts of the country, rapid urbanization processes have been taken place in the district in the recent past. However, the district reports high case incidence rates of dengue not only from urban areas but also from semiurban and rural areas. Out of 29 Medical Officer of Health (MOH) areas in Kurunegala district, 2 have been classified as priority high risk MOH areas and 12 as high risk for dengue. Vector control in the district is mainly by source reduction, application of insecticides, public health education, and legislations. The present study aimed to assess spatial and temporal distribution of dengue vector mosquitoes in some selected localities of the Kurunegala district of Sri Lanka over a three-year period and to predict vector prevalence with respect to changing weather parameters.

## 2. Methods

### 2.1. Study Sites

**Figure 1:**
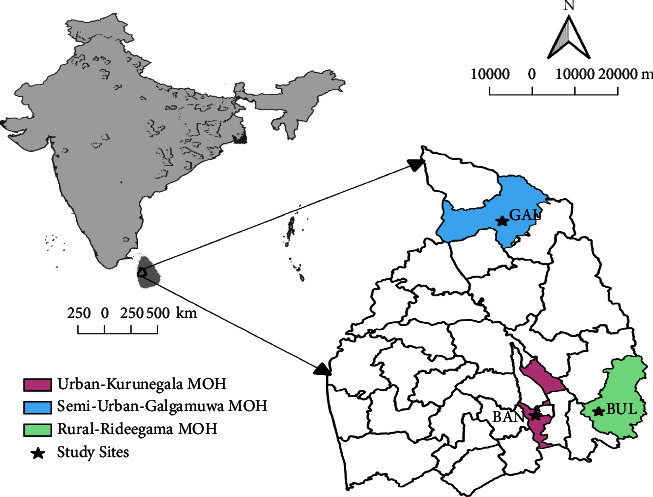
Map of study areas in Kurunegala districts. Eggs and larvae of *Aedes aegypti* and *Ae. albopictus* were collected from three localities: urban Bandaranayakapura in Kurunegala MOH area, semiurban Galgamuwa in Galgamuwa MOH area, and rural Buluwala in Rideegama MOH area.

The study was conducted in three selected localities in the Kurunegala district (7°45′ N, 80° 15′ E) which is located in the North-Western Province of Sri Lanka covering 4812.7 km^2^ with a population of approximately 1,676,000 living in nearly 439,065 households. About 32.6% of the population depends on agriculture-related employment. Average maximum and minimum temperatures were approximately 32.83°C and 23.43°C, respectively, during the study period. Annual accumulated rainfall and average humidity were approximately 164.65 mm and 67%. The three selected localities i.e., urban Bandaranayakapura (BAN) in Kurunegala MOH area, semiurban Galgamuwa (GAL) in Galgamuwa MOH area, and rural Buluwala (BUL) in Rideegama MOH area (Figure 1), were from dengue high risk areas based on the dengue and dengue epidemic prevalence data for the previous five-year period (2013–2018). The study was carried out only in residential premises (premises with care of house owners) of all study sites. Bandaranayakapura (96 km^2^) is an urbanized locality with about 600 households with water supply, sanitation facilities, electricity services, and rubbish collection within an area of. Galgamuwa (278.4 km^2^) is a suburban locality with about 500 households, poor sanitary facilities, and no municipal water supply. Buluwala (220 km^2^) is a rural area with 400 householders, dense vegetation, and mountains.

### 2.2. Egg Surveys

Monthly ovitrap surveys were conducted from January to December 2019 in 50 randomly selected house premises at each study site except for urban BAN area where the time period of the survey was extended up to December 2021. Following WHO guidelines, mosquito eggs were collected using standard ovitraps which were made with black plastic cups (8 cm height × 7 cm diameter) filled with dechlorinated tap water.

A rectangular filter paper strip (3 × 22 cm) was placed inside the cup as the oviposition substrate. Two traps were kept at each house premise and the filter paper strips were labeled with the date, house number, and the location. One ovitrap was kept outdoor 5–10 m away from the house (depending on the size of the garden) and was labeled as “A.” The other trap “B” was kept inside the house. Filter paper strip of each trap was replaced with a new one once in every 5 days, and the removed filter papers were brought to the laboratory at the entomological surveillance unit, RDHS office, Kurunegala. In the laboratory, filter paper strips with the eggs were air-dried and stored at room temperature. They were examined under a stereomicroscope and the eggs of the two dengue vector species were identified using the shape and the size. To confirm the identity, randomly selected eggs of the two types were reared in separate containers and the larvae were identified using the key introduced by Tanaka and Mizusawa [17]. Number of eggs of the two dengue vector species were counted and recorded separately. Ovitrap index (OVI) was calculated as the percentage of positive ovitraps, and the egg density index (EDI) was calculated as the average number of eggs per trap for each survey.

### 2.3. Larval Surveys

Monthly larval surveys were conducted from January to December 2019 at each study sites except for BAN area where the survey was continued up to December 2021. Following the systematic sampling approach, every 6^th^ premise in the inspection route of the locality was chosen for sampling and a minimum of 100 premises was selected from each study site. At each premise, all the potential containers in both indoor and outdoor areas were examined, and sampling was performed from all positive containers. Larvae were collected using dippers and pipets (50 ml) depending on the nature of the container. Collected larvae were transferred to labeled plastic vials and transported to the laboratory for further analysis. All the larvae were reared to 3^rd^ and 4^th^ instar levels for identification using standard larval identification keys [17]. Larval indices, i.e., Premise/house Index (PI/HI), Container Index (CI), and Breteau Index (BI) were calculated for both vector species to determine the larval abundance at each site [2, 18].

### 2.4. Meteorological Data

Maximum and minimum daily temperature, daily rainfall, and daily relative humidity for all study sites were obtained from the Department of Meteorology, Colombo, Sri Lanka.

### 2.5. Statistical Analyses

Data obtained were formatted for layouts using MS-Excel. As a preliminary analysis, meteorological data were subjected to the Anderson–Darling Normality Test at a significance level of 5% using Minitab 16. When distributions were not in accordance with the criteria of normality, data were transformed into Log10 to obtain a normal distribution. Study results were descriptively summarized using frequencies, proportions, means, and standard deviations. The larval indices (PI, CI, and BI) and egg indices (OVI and EDI) of urban, semiurban, and rural settings were comparatively analyzed by mean comparisons using the independent sample *t*-test and one-way ANOVA.

#### 2.5.1. Correlation and Regression Analyses

Correlation analysis was performed using Spearman's rank correlation analysis. Relationship between the indices and the weather parameters (monthly rainfall (mm), minimum and maximum temperature (°C), and relative humidity (%)) in different sites was analyzed. Spatial autocorrelations were tested using Moran's I statistic, and the variables selected for the final model satisfied the independency at 5% significant level. Correlation analysis was done between the selected meteorological variable with different time lags (no lag, 1-week lag, 2-week lag etc.). The lag period that had the highest correlation with the index was used to create the models.

Stepwise multiple regression analysis (SMR) was done to develop a prediction model to evaluate the association between weather variables with the larval indices (PI, CI and BI) and egg indices (OVI and EDI). A forecasting regional model (FRM) was then developed and evaluated to predict the larval indices. The predicted larval indices could then be used to forecast possible dengue outbreaks by predicting the patterns of larval abundance of dengue vectors. The general model was based on the following equation:

(1) $$\begin{array}{c} y=\beta_{0}+\beta_{i}X_{i}+\ldots +\beta_{n}X_{n}\text{,} \end{array}$$

where *y* is the estimated index value, *β*_0_ is a constant, *β*_*i* _ are coefficients of environmental variables *X*_*i*_.

*R*-squared and *F*-test were used to statistically measure how close the data are to the fitted regression line. It is also known as the coefficient of determination, or the coefficient of multiple determinations for multiple regressions. To validate models, actual and predicted values were used.

## 3. Results

### 3.1. Abundance of Dengue Vector Species in Different Urban Settings

#### 3.1.1. Egg Survey

A total of 24,403 eggs were collected from the ovitrap survey. Out of 3550 ovitraps placed, 1535 were positive for *Aedes* eggs in the three study sites during January to December 2019 (urban = 421 (27.46%); semiurban = 513 (33.42%); rural = 601 (39.15%)). *Aedes aegypti* eggs were less abundant (*n* = 497 (2.03%)) while *Ae. albopictus* eggs were dominant (*n* = 23,872 (97.82%)) in positive ovitraps. Mixed infestation of both *Ae. aegypti* and *Ae. albopictus* was not common (*n* = 34, 0.14%).

*Aedes aegypti* eggs were present only in urban and semiurban areas, and OVI at urban site was significantly higher than that at semiurban site (*p*=0.001) (Table 1). *Aedes albopictus* eggs were predominant in all study sites. Although the highest number of eggs were received from rural followed by semiurban sites, the OVI of *Ae. albopictus* was not significantly different between study sites (*p*=0.456). *Aedes aegypti* eggs showed a significantly higher abundance in indoor than outdoor collections (*p*=0.012), and *Ae. albopictus* eggs were more abundant in outdoor collections (*p*=0.001) (Table 1).

#### 3.1.2. Larval Survey

During the period January to December 2019, a total of 3693 house premises were inspected. Out of them, 240 (6.4%) premises were positive for dengue vector larvae. A total of 2172 *Aedes* larvae were collected from these premises [*Ae. aegypti* larvae *n* = 94 (4.33%); *Ae. albopictus* larvae *n* = 2078 (95.67%)]. *Aedes aegypti* larvae were obtained only from the urban locality (*n* = 94, 4.33%) while *Ae. albopictus* larvae were reported from all three study sites [Urban: 531 (24.4%); semi-urban: 567 (26.1%); rural: 981 (45.16%)]. However, the larval indices PI, CI and BI for *Ae. albopictus* were not significantly different among the study sites (*p*=0.234) (Table 2).

### 3.2. Seasonal Fluctuation of OVI and BI

**Figure 2:**
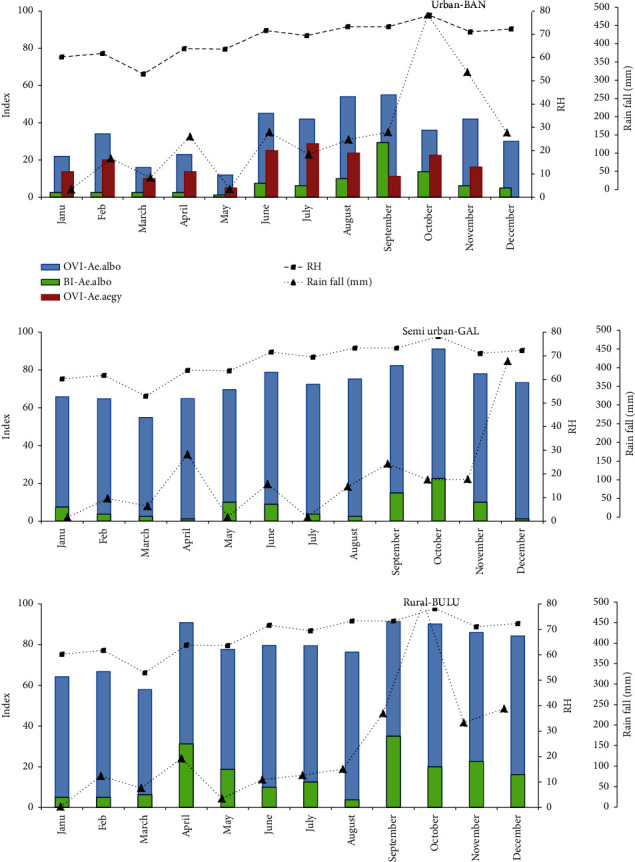
Seasonal variation of the ovitrap index (OVI) of *Aedes aegypti* in urban Bandaranayakapura (BAN)and OVI and the larval Breteau index (BI) of *Ae. albopictus* in BAN, semiurban Galgamuwa (GAL), and rural Buluwala (BUL) against rainfall and relative humidity (RH) from January 2019 to December 2019.

Larval numbers of BAN *Ae. aegypti* were too low to show the seasonal fluctuation of BI. Monthly variations of OVI for BAN *Ae. aegypti* and, OVI and BI for *Ae. albopictus* from all three study sites were plotted against the rain fall and relative humidity for the period January to December 2019 (Figure 2). An increasing trend could be observed both in OVI and BI with the rainfall in all three areas. However, a decline of BI was observed with heavy rain falls in September-November, probably due to flush over of the breeding sites (Figure 2).

### 3.3. Correlation of OVI and BI with Weather Parameters

Sessional variation of egg and larval indices of both species were analyzed against rain fall (RF), relative humidity (RH), and temperature data for the period January to December 2019. For *Ae. aegypti*, no correlation was obtained between the indices and weather parameters since the egg and larval numbers were very low. All the variables indicated Moran's *I* index was close to zero. Container Index and Premise Index of *Ae. albopictus* showed a significant spatial autocorrelation across the study period, while BI indicated a nonsignificant (*p* > 0.05) and weak spatial autocorrelation (Moran's *I* = 0.125). We found BI to be suitable for developing the prediction model. Ovitrap Index of *Ae. albopictus* showed lower spatial autocorrelation (Moran's *I* = 0.002) across the study period. Therefore, development of a prediction model for mosquito abundance using ovitrap collections was suggested. For *Ae. albopictus*, OVI, PI, CI, and BI correlated with both the RF and RH only for the urban site. However, correlations were strongly positive between OVI and RH (*r* = 0.680, *p*=0.027), and between BI and RF (*r* = .970, *p*=0.002). Therefore, it was decided to continue the study in urban (BAN) area for further two years to develop prediction models for these indices.

### 3.4. Prediction Model for OVI

When considering three-year data from Jan 2019 to Dec 2021, the correlation between OVI and RH was still significant and was strongly positive (*r* = 0.786, *p*=0.001). The association between OVI and RH was then evaluated in 1 week, 2 week, 3 week, 4 week, and 8 week lag periods. Significant positive correlations were obtained for 4 week (*r* = 0.610, *p*=0.002) and 8 week (*r* = 0.624, *p*=0.001) lag periods.

Linear regression analysis was fitted and a prediction model was developed using OVI and RH with no lag period (*R*^2^ (sq) = 77.24%; *F* = 23.7; model: OVI predicted = −28.9 + 0.931^*∗*^ RH; RMSE = 3.47), with 1 month lag period (*R*^2^ (sq) = 70.21%; *F* = 57.23; model: OVI predicted = 15.1 + 0.528^*∗*^ Lag 1 month RH; RMSE = 2.01), with 2-month lag period (OVI predicted = 14.7 + 0.536^*∗*^ Lag 2 month RH; RMSE = 2.28).

**Figure 3:**
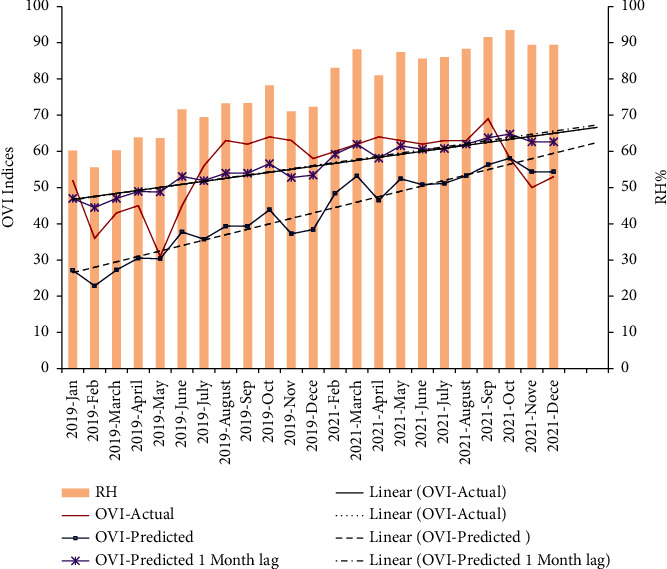
Actual trend and the model-generated predicted trend (without lag and with a 1-month lag) for oviposition index (OVI) variation of *Aedes albopictus* in relation to relative humidity (RH) in urban Bandaranayakapura (BAN) area.

Since the lowest root mean square error (RSME) value (2.01) was with actual and lag 1-month predicted values, a model was generated for RH/actual OVI and RH/1-month lag OVI for urban (BAN) *Ae. albopictus* (Figure 3). It is interesting to see that the linear predicted 1-month lag OVI line directly overlaps with the linear actual OVI line.

### 3.5. Prediction Model for BI

Three-year data (2019–2021) showed a positive correlation between BI and RF (*r* = 0.789, *p*=0.002) for BAN *Ae. albopictus*. The association between BI and RF was evaluated with actual time RF and lag periods after RF. Significant correlations were obtained up to the 3-week lag period (*r* = 0.970 for no lag, 0.495 for 1-week lag, 0.381 for 2-week lag, and 0.306 for 3-week lag).

**Figure 4:**
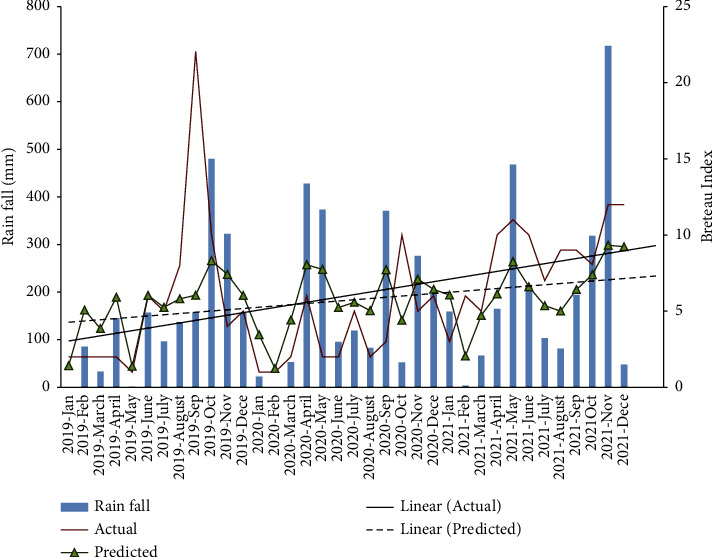
Actual trend and the model generated predicted trend of Breteau index (BI) for *Ae. albopictus* with rain fall in urban Bandaranayakapura (BAN) area.

Linear regression analysis was fitted, and a prediction model was developed using BI and RF with no lag period (*R*^2^ (sq) = 86.3%; *F* = 53.12; *R*^2^ (pred) = 63.12%; model: Log10 (BI) = 0.153 + 0.286∗ Log10 (RF); RMSE = 1.49) (Figure 4).

## 4. Discussion

Dengue is considered the most prevalent vector borne disease in tropics. Also, it is reportedly sensitive to environmental changes such as climate change and urbanization. Spatial distribution of vectors along urban-rural gradients, with *Ae. aegypti* preferring urban and *Ae. albopictus* preferring rural environments has been observed previously [19, 20], and the present results further confirm this observation. During the present study *Ae. aegypti* was found in the urban area, and to a lesser extent, in the semiurban area but not in the rural area. *Aedes albopictus* was dominating from rural to the urban area indicating its invasion of urban areas. Higher adaptability and invasive behavior of this species have been reported elsewhere [15, 21]. Our results confirm previous findings that *Ae. aegypti* prefers indoor breeding to outdoor while *Ae. albopictus* prefers both indoor and outdoor breeding [12, 22, 23]. Both the feeding and breeding behavior of *Ae. aegypti* are more or less limited to domestic environments whereas the activity pattern of *Ae. albopictus* has a wider range as it equally prefers to feed on both wild animals and humans and is more adaptive to breed in variety of environments [3, 19, 20, 24]. As far as the transmission of the disease is concerned, both these vectors are equally important, as DENV has been isolated from both these vectors [25, 26]. Some studies have suggested that the dengue virus transmission risk is higher with *Ae. albopictus* due to its wider distribution [27]. High prevalence of both these vectors has been reported during the 2017-2018 dengue outbreak in the Kurunegala district (Entomological report data, RDHS, Kurunegala).

Sri Lanka receives southwest monsoon rains during May to September and northeast monsoon rains during December to February. Surendran et al. [28] has reported egg density peaks corresponding to increased precipitation in monsoonal periods from the northern Jaffna district of Sri Lanka. Kurunegala district is situated in northwest region and hardly receives monsoon rains. The district receives rains mainly from intermonsoons, which occur throughout the country during March to April and October to November periods and a mild rainfall from conventional rains. All three study sites reported increased precipitation towards the end of the year. Studies have emphasized that an OVI above 10% for *Aedes* vector species is an indication of a possible risk of dengue outbreak, provided that dengue positive cases are present in the area [29]. If so, all three study sites used for the present study were at dengue outbreak-risk state if the virus is available. Entomological surveillances are mainly based on different larval indices and the House Index (HI-percentage of houses positive for larvae) and the Breteau index (BI-number of positive containers per 100 houses) are the most widely used larval indices [4]. However, discriminating thresholds have never been determined for larval indices for dengue fever transmission. Values of HI > 1% and BI > 5 have been proposed to indicate high risk levels for yellow fever transmission [4]. If a similar level of discrimination is applied for dengue, our study areas are at high risk of dengue if the virus is available.

Weather factors such as rainfall (RF), temperature, and relative humidity (RH) have been widely studied for their usability in predicting dengue incidence trends [30, 31]. Previous workers have reported that temperature and humidity have significant associations with the dengue incidence [32–36]. It has been suggested that RH can affect larval density by extending adult mosquito survival [37]. Wu et al. [32] and Campbell et al. [34] identified RF as having a strong positive correlation with dengue incidence. There can be several factors, other than weather, which may act critically in determining spatial distribution of dengue mosquitoes. Urbanization, waste management, sanitary facilities, community awareness, and vector control strategies are some of these. However, some researchers have shown that the value of entomological indices as predictors of *Aedes*-borne disease outbreaks is inconclusive and limited due to the complex epidemiology of dengue, which involves dynamic interplay of multiple factors such as herd immunity within a population, distinct serotypes of the virus, intervention programs, mosquito-human interaction, and mosquito-virus interaction [38, 39]. Nevertheless, significant influence of meteorological factors on the development, survival, density, and oviposition rate of the vector mosquitoes, expressed as larval [40], pupal [41], and adult indices [38] can certainly make an indication on dengue disease transmission.

Forecasting vector abundance with weather variables have been attempted by several workers. Moore et al. [42] in Puerto Rico and Pontes et al. [40] in Fortaleza, Brazil, used temporal graphics to compare seasonal fluctuation of RF, *Aedes* larval indices, and dengue incidence. They observed a strong relationship between larval indices and RF patterns. Present study demonstrated that OVI, PI, CI, and BI of *Ae. albopictus* correlates with both the RF and RH in urban areas. Strong positive association between OVI and RH and between BI and RF for *Ae. albopictus* in urban areas compelled us to continue sampling for two more years and to develop prediction models to forecast OVI and BI by using respective RH and RF data. However, as it has been reported previously, heavy precipitation flushed the water containers removing mosquito larvae away from breeding sites resulting a decline of BI [43, 44].

A significant relationship, giving the lowest RSME value (2.01), was observed between RH and *Ae. albopictus* OVI with a lag of 1 month in urban area. In the generated model, the linear predicted 1-month lag OVI line directly overlaps with the linear actual OVI line showing the accuracy in predicting. Several workers have shown the influence of RH on adult mortality [45, 46], egg maturity process, and egg laying inducement in *Aedes* mosquitoes [47]. Prediction model for BI against RF gave a low RMSE (1.49) indicating that the model can be successfully used to forecast mosquito populations with respect to RF changes in urban areas. It was observed that BI significantly correlates with RF even after a period of up to three weeks, for *Ae. Albopictus*. Assessment of RMSE and *R* square values suggested that our model can provide accurate predictions on the abundance of *Ae. Albopictus*. Therefore, these models can be successfully utilized to predict vector prevalence in urban sites, and successful vector control interventions can be adopted during the lag periods to minimize disease incidence.

## 5. Conclusions

*Aedes aegypti* population was mainly found in urban area, and to a lesser extent, in semiurban area. It was not found in rural areas. *Aedes albopictus* was present in all three areas with a preference from rural to urban but dominating even the urban settings. *Aedes aegypti* preferred indoor breeding while *Ae. albopictus* preferred both indoor and outdoor. Strong correlations were observed between the ovitrap index (OVI) and the relative humidity (RH) and between Breteau index (BI) and the rainfall (RF). Prediction models were developed using BI and RF with no lag period and using OVI and RH with 1 month lag period. These two models will be able to detect population dynamics of *Ae. albopictus* vector mosquitoes in urban settings allowing to predict possible dengue outbreaks.

## Figures and Tables

**Table 1:** Ovitrap positivity (OVI ± SE) and egg density (EDI ± SE) of *Aedes aegypti* and *Ae. albopictus* in three study sites.

Variable		Urban-BAN			Semiurban-GAL			Rural-BUL		
		Total no. of eggs	OVI	EDI	Total no. of eggs	OVI	EDI	Total no. of eggs	OVI	EDI
*Ae. aegypti*	Indoor	270	15.67^a^ ± 3	1.1^e^ ± 0.3	18	1.2^a^ ± 0.7	0.43^f^ ± 0.2	^*∗*^	^*∗*^	^*∗*^
	Outdoor	209	9.67^b^ ± 1	0.9^e^ ± 0.1	^*∗*^	^*∗*^	^*∗*^	^*∗*^	^*∗*^	^*∗*^

*Ae. albopictus*	Indoor	2885	33.83^d^ ± 5	12^g^ ± 9	3050	47^d^ ± 2	12^g^ ± 2	3851	38.67^d^ ± 2	12.3^g^ ± 8
	Outdoor	4126	34.67^d^ ± 4	17.2^g^ ± 11	3570	53.2^d^ ± 2	0.4^f^ ± 0.03	7310	60.00^e^ ± 3	18.43^g^ ± 2

^*∗*^Eggs were not present. BAN, Bandaranayakapura; GAL, Galgamuwa; BUL, Buluwala. Different letters indicated statistically significant difference between variables using the ANOVA test.

**Table 2:** Larval indices (Mean ± SE) for *Aedes aegypti* and *Ae. albopictus* collected from the study sites.

Species	Urban-BAN				Semi-urban-GAL				Rural-BUL			
	No. of larvae	PI	CI	BI	No. of larvae	PI	CI	BI	No. of larvae	PI	CI	BI
*Ae. aegypti*	94	1.3 ± 0.5	2 ± 1.1	1.4 ± 0.7	^*∗*^	^*∗*^	^*∗*^	^*∗*^	^*∗*^	^*∗*^	^*∗*^	^*∗*^
*Ae. albopictus*	531	7.9 ± 1.2	9.3 ± 1.6	4.1 ± 1.9	567	4.9 ± 1.4	8.7 ± 1.4	5.9 ± 1.1	981	11.3 ± 1.5	14.3 ± 1.6	12.2 ± 1.7

^*∗*^Larvae not present. PI, premise index; CI, container index; BI, Breteau index; BAN, Bandaranayakapura; GAL, Galgamuwa; BUL, Buluwala.

## Data Availability

The data used to support the findings of this study are available from the corresponding author upon request.

## References

[1] World health organization (2014). *Yellow Fever. Rapid Field Entomological Assessment During Yellow Fever Outbreaks in Africa. Methodological Field Approaches for Scientists with a Basic Background in Entomology*.

[2] World health organization (2019). *Dengue and Severe Dengue*.

[3] Higa Y. (2011). Dengue Vectors and their spatial distribution. *Tropical Medicine and Health*.

[4] World Health Organization (2011). *Regional Office for South-East Asia. Comprehensive Guideline for Prevention and Control of Dengue and Dengue Haemorrhagic Fever. Revised and expanded edition*.

[5] Who (2009). *Dengue Guidelines for Diagnosis, Treatment, Prevention and Control*.

[6] Li Y., Kamara F., Yao L. (2014). Urbanization increases *Aedes albopictus* larval habitats and accelerates mosquito development and survivorship. *PLoS Neglected Tropical Diseases*.

[7] Tsai P J., Teng H. J. J. (2016). Role of *Aedes aegypti* (Linnaeus) and *Aedes albopictus* (Skuse) in local dengue epidemics in Role of *Aedes aegypti* (Linnaeus) and *Aedes albopictus* (Skuse) in local dengue epidemics in Taiwanaiwan. *BMC Infectious Diseases*.

[8] Lounibos L. P. P. (2002). Invasions by insect vectors of human disease. *Annual Review of Entomology*.

[9] Surendran S. N., Jayadas T. T. P., Thiruchenthooran V. (2021). Aedes larval bionomics and implications for dengue control in the paradigmatic Jaffna peninsula, northern Sri Lanka. *Parasites & Vectors*.

[10] Dalpadado C. P. R. D., Amarasinghe L. D. Abundance and distribution pattern of *Aedes aegypti* and *Aedes albopictus* in selected urban, sub-urban and rural areas of Gampaha District, Sri Lanka.

[11] Weeraratne T. C., B Perera M. D., Karunaratne S. P., Perera M. D. B., Mansoor M. A. C. M., Karunaratne S. H. P. P. (2013). Prevalence and breeding habitats of the dengue vectors *Aedes aegypti* and *Aedes albopictus* (Diptera: Culicidae) in the semi-urban areas of two different climatic zones in Sri Lanka. *International Journal of Tropical Insect Science*.

[12] Wijegunawardana N. D. A. D., Gunawardene Y. I. N. S., Chandrasena T. G. A. N., Dassanayake R. S., Udayanga N. W. B. A. L., Abeyewickreme W. D. (2019). Evaluation of the Evaluation of the Effects of *Aedes* Vector Indices and Climatic Factors on Dengue Incidence in Gampaha District, Sri Lanka. *BioMed Research International*.

[13] Dissanayake D. S., Wijekoon C. D., Wegiriya H. C. (2021). The effect of breeding habitat characteristics on the larval abundance of Aedes vector mosquitoes (Diptera: Culicidae) in three localities, galle district, Sri Lanka. *Journal of Entomology*.

[14] Gubler D. J. J. (2011). Dengue, urbanization and globalization: the unholy trinity of the 21st century. *Tropical Medicine and Health*.

[15] Khormi H. M., Kumar L., Elzahrany R. A. (2014). Regression model for predicting adult female *Aedes aegypti* based on meteorological variables: a case study of jeddah, Saudi arabia. *Journal of Earth Science & Climatic Change*.

[16] Epidemiology unit Ministry of Health Sri Lanka (2020). Dengue, Disease Surveillance Trends. http://www.epid.gov.lk.

[17] Tanaka K. K., Mizusawa K. (1979). Saugstad ESA revision of the adult and larval mosquitoes of Japan (including the ryukyu archipelago and the ogasawara islands) and korea (Diptera: Culicidae). *Contributions of the American Entomological Institute*.

[18] Favier C., Degallier N., Ribeiro Vilarinhos P. D. T. (2006). Effects of climate and different management strategies on *Aedes aegypti* breeding sites: a longitudinal survey in Brasilia (DF, Brazil). *Tropical Medicine and International Health*.

[19] Bagny L., Delatte H., Fontenille D. (2009). Aedes (Diptera: Culicidae) vectors of arboviruses in Mayotte (Indian Ocean): distribution area and larval habitats. *Journal of Medical Entomology*.

[20] Caputo B., Ienco A., Petrarca V. (2012). The “auto-dissemination” approach: a novel concept to fight *Aedes albopictus* in urban areas. *PLoS Neglected Tropical Diseases*.

[21] Bonizzoni M., Gasperi G., Chen X., James A. A., Chen X., James A. A. (2013). The invasive mosquito species *Aedes albopictus*: current knowledge and future perspectives. *Trends in Parasitology*.

[22] Agha S. B., Chepkorir E., Ambala P. (2017). Vector competence of populations of *Aedes aegypti* from three distinct cities in Kenya for chikungunya virus. *PLoS Neglected Tropical Diseases*.

[23] Nirmani M. D., Perera K. L. N. S., Galhena G. H., Perera K. L. N. S., Galhena G. H. (2019). Use of ovitrap surveillance to assess dengue outbreak risks in selected dengue endemic areas in Sri Lanka. *Sri Lankan Journal of Biology*.

[24] Delatte H., Dehecq J., Thiria J. (2008). Geographic distribution and developmental sites of *Aedes albopictus* (Diptera: Culicidae) during a Chikungunya epidemic event. *Vector Borne and Zoonotic Diseases*.

[25] Kusumawathie P. H. D., Fernando W. P. H. D., Fernando W. P. (2003). Breeding habitats of *Aedes aegypti* Linnaeus and *Aedes albopictus* Skuse in a dengue transmission area Kandy, Sri Lanka. Sri Lanka. *Ceylon Journal of Medical Science*.

[26] Tewari S. C., Thenmozhi V., Katholi C. R. (2004). Dengue vector prevalence and virus infection in a rural area in South India. *Tropical Medicine and International Health*.

[27] Hapugoda M. D., Gunasekera M. B., Silva N. R., Gunasena S., Prithimala L. D., Dayanath M. Y. D. (2003). Detection of dengue virus in *Aedes albopictus* mosquitoes by reverse transcription polymerase-chain reaction-liquid hybridization (RT-PCR-LH) based assay. *The Bulletin of the Sri Lanka College of Microbiologists*.

[28] Surendran S. N., Kannathasan S., Kajatheepan A., Jude P. J. (2007). Chikungunya-type fever outbreak: some aspects related to this new epidemic in Jaffna district, northern Sri Lanka. *Tropical Medicine and Health*.

[29] Braks M. A. H., Honorio N. A., Lourenço-De-Oliveira R., Juliano S A, Lounibos L. P., Lounibos L. P. (2003). Convergent habitat segregation of aedes aegypti and aedes albopictus(diptera: culicidae) in southeastern brazil and florida. *Journal of Medical Entomology*.

[30] Ibarra A. M. S., Ryan S. J., Beltrán E. (2013). Dengue dengue vector dynamics (aedes aegypti) influenced by climate and social factors in ecuador: implications for targeted control. *PLoS One*.

[31] Bowman L. R., Runge-Ranzinger S., McCall P. J., Runge-Ranzinger S., McCall P. J. (2014). Assessing the relationship between vector indices and dengue transmission: a systematic review of the evidence. *PLoS Neglected Tropical Diseases*.

[32] Wu P. C., Guo H. R., Lung S. C. (2007). Weather as an effective predictor for occurrence of dengue fever in Taiwan. *Acta Tropica*.

[33] Barrera R., Amador M., MacKay A. J., MacKay A. J. (2011). Population dynamics of *Aedes aegypti* and dengue as influenced by weather and human behavior in San Juan, Puerto Rico. *PLoS Neglected Tropical Diseases*.

[34] Campbell K. M., Lin C. D., Iamsirithaworn S. (2013). The complex relationship between weather and dengue virus transmission in Thailand. *The American Journal of Tropical Medicine and Hygiene*.

[35] Vu H. H., Okumura J., Hashizume M. (2014). Regional differences in the growing incidence of dengue fever in Vietnam explained by weather variability. *Tropical Medicine and Health*.

[36] Yang C. F., Hou J. N., Chen T. H. (2014). Discriminable roles of *Aedes aegypti* and *Aedes albopictus* in establishment of dengue outbreaks in Taiwan. *Acta Tropica*.

[37] Sang S., Yin W., Wang C. (2014). Predicting local dengue transmission in Guangzhou, China, through the influence of imported cases, mosquito density and climate variability. *PLoS One*.

[38] Rodriguez-Figueroa L., Rigau-Perez J. G., Suarez E. L., Reiter P. G., Suarez E. L., Reiter P. (1995). Risk factors for dengue infection during an outbreak in Yanes, Puerto Rico in 1991. *The American Journal of Tropical Medicine and Hygiene*.

[39] Scott T. W., Morrison A. C., Takken W., Scott T. W. (2004). *Aedes aegypti* density and the risk of dengue-virus transmission. *Ecological Aspects for Application of Genetically Modified Mosquitoes*.

[40] Pontes R. J., Spielman A., Oliveira-Lima J. W., Hodgson J. C., Freeman J. (2000). Vector densities that potentiate dengue outbreaks in a Brazilian city. *The American Journal of Tropical Medicine and Hygiene*.

[41] Focks D. A., Hayes J., Brenner R. J. (2000). Transmission thresholds for dengue in terms of *Aedes aegypti* pupae per person with discussion of their utility in source reduction efforts. *The American Journal of Tropical Medicine and Hygiene*.

[42] Moore C. G., Cline B. L., Ruiz-Tibén E. (1978). *Aedes aegypti* in Puerto Rico: environmental determinants of larval abundance and relation to dengue virus transmission. *The American Journal of Tropical Medicine and Hygiene*.

[43] Focks D., Barrera R. (2007). Dengue transmission dynamics: assessment and implications for control. *Report of the Scientific Working Group Meeting on Dengue*.

[44] Karim M. N., Munshi S. U., Anwar N. (2012). Climatic factors influencing dengue cases in Dhaka city: a model for dengue prediction. *Indian journal of medical research*.

[45] Christophers S. R. (1960). *Aedes aegypti (L.) the Yellow Fever Mosquito*.

[46] Micieli M. V., Campos R. E. V., Campos R. E. (2003). Oviposition activity and seasonal pattern of a population of Aedes (Stegomyia) aegypti (L.) (Diptera: Culicidae) in Subtropical Argentina. *Memorias do Instituto Oswaldo Cruz*.

[47] Estallo E. L., Lamfri M. A., Scavuzzo C. M. (2008). Models for predicting *Aedes aegypti* larval indices based on satellite images and climatic variables. *Journal of the American Mosquito Control Association*.

